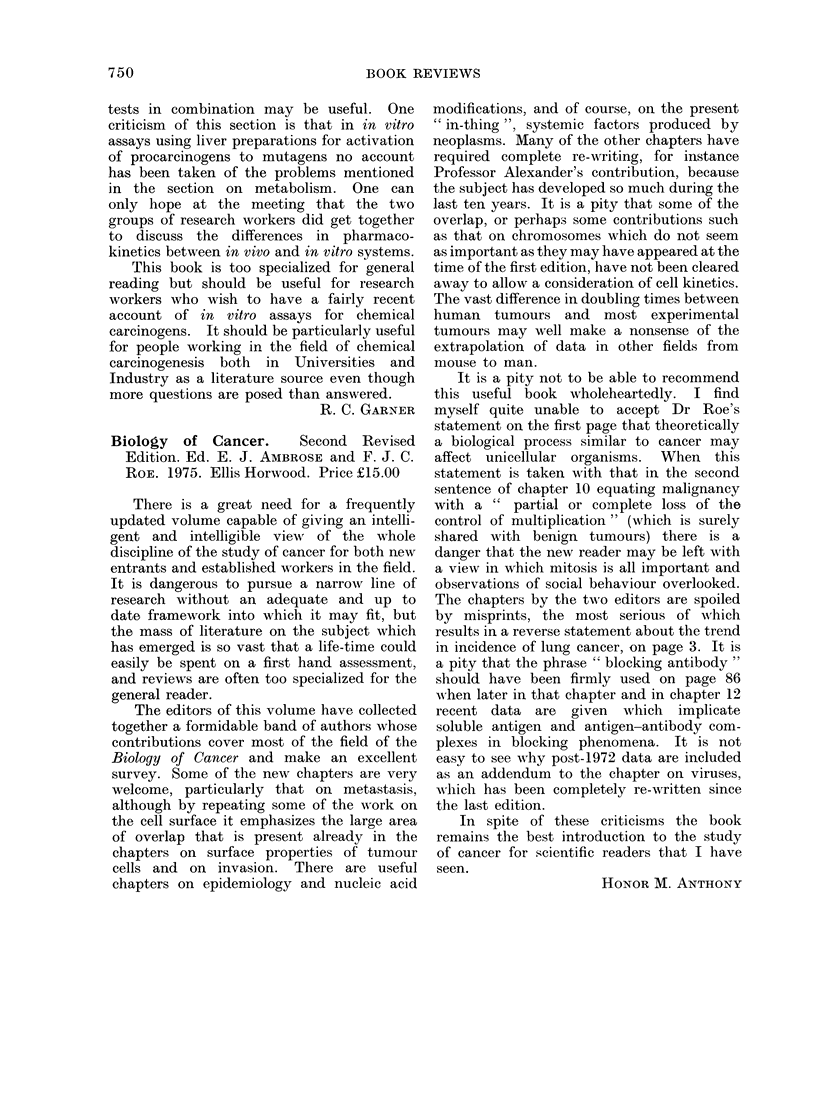# Biology of Cancer

**Published:** 1975-12

**Authors:** Honor M. Anthony


**Biology of Cancer.** Second Revised Edition. Ed. E. J. Ambrose and F. J. C. Roe. 1975. Ellis Horwood. Price £15.00

There is a great need for a frequently updated volume capable of giving an intelligent and intelligible view of the whole discipline of the study of cancer for both new entrants and established workers in the field. It is dangerous to pursue a narrow line of research without an adequate and up to date framework into which it may fit, but the mass of literature on the subject which has emerged is so vast that a life-time could easily be spent on a first hand assessment, and reviews are often too specialized for the general reader.

The editors of this volume have collected together a formidable band of authors whose contributions cover most of the field of the *Biology of Cancer* and make an excellent survey. Some of the new chapters are very welcome, particularly that on metastasis, although by repeating some of the work on the cell surface it emphasizes the large area of overlap that is present already in the chapters on surface properties of tumour cells and on invasion. There are useful chapters on epidemiology and nucleic acid modifications, and of course, on the present " in-thing ", systemic factors produced by neoplasms. Many of the other chapters have required complete re-writing, for instance Professor Alexander's contribution, because the subject has developed so much during the last ten years. It is a pity that some of the overlap, or perhaps some contributions such as that on chromosomes which do not seem as important as they may have appeared at the time of the first edition, have not been cleared away to allow a consideration of cell kinetics. The vast difference in doubling times between human tumours and most experimental tumours may well make a nonsense of the extrapolation of data in other fields from mouse to man.

It is a pity not to be able to recommend this useful book wholeheartedly. I find myself quite unable to accept Dr Roe's statement on the first page that theoretically a biological process similar to cancer may affect unicellular organisms. When this statement is taken with that in the second sentence of chapter 10 equating malignancy with a " partial or complete loss of the control of multiplication " (which is surely shared with benign tumours) there is a danger that the new reader may be left with a view in which mitosis is all important and observations of social behaviour overlooked. The chapters by the two editors are spoiled by misprints, the most serious of which results in a reverse statement about the trend in incidence of lung cancer, on page 3. It is a pity that the phrase " blocking antibody " should have been firmly used on page 86 when later in that chapter and in chapter 12 recent data are given which implicate soluble antigen and antigen–antibody complexes in blocking phenomena. It is not easy to see why post-1972 data are included as an addendum to the chapter on viruses, which has been completely re-written since the last edition.

In spite of these criticisms the book remains the best introduction to the study of cancer for scientific readers that I have seen.

Honor M. Anthony